# Stress response as implemented by hibernating ribosomes: a structural overview

**DOI:** 10.1111/febs.14968

**Published:** 2019-07-15

**Authors:** Donna Matzov, Anat Bashan, Mee‐Ngan F. Yap, Ada Yonath

**Affiliations:** ^1^ Structural Biology Weizmann Institute. Rehovot Israel; ^2^ Biochemistry and Molecular Biology Saint Louis University School of Medicine St. Louis MO USA; ^3^ Microbiology and Immunology Northwestern University Chicago IL USA

**Keywords:** 100S, hibernation, ribosome, single particle cryo‐EM

## Abstract

Protein synthesis is one of the most energy demanding cellular processes. The ability to regulate protein synthesis is essential for cells under normal as well as stress conditions, such as nutrient deficiencies. One mechanism for protein synthesis suppression is the dimerization of ribosomes into hibernation complexes. In most cells, this process is promoted by the hibernating promoting factor (HPF) and in a small group of Gram‐negative bacteria (γ‐proteobacteria), the dimer formation is induced by a shorter version of HPF (HPF^short^) and by an additional protein, the ribosome modulation factor. In most bacteria, the product of this process is the 100S ribosome complex. Recent advances in cryogenic electron microscopy methods resulted in an abundance of detailed structures of near atomic resolutions 100S complexes that allow for a better understanding of the dimerization process and the way it inhibits protein synthesis. As ribosomal dimerization is vital for cell survival, this process is an attractive target for the development of novel antimicrobial substances that might inhibit or stabilize the complex formation. As different dimerization processes exist among bacteria, including pathogens, this process may provide the basis for species‐specific design of antimicrobial agents. Here, we review in detail the various dimerization mechanisms and discuss how they affect the overall dimer structures of the bacterial ribosomes.

**Abbreviations**
Anti‐SD: anti Shine–Dalgarno
A‐tRNA: A‐site bound tRNA
cryo‐EM: cryogenic electron microscopy
CTD: C‐terminal domain
E‐tRNA: tRNA located at the ribosome’s exit site
HPF^long^: long version of the hibernation promoting factor
HPF ^short^: short version of the hibernation promoting factor
HPF: hibernation promoting factor
LSU: Large subunit of the ribosome
NTD: N‐terminal domain
PBC: platform binding center
PTC: peptidyl transferase center
P‐tRNA: P‐site bound tRNA
RMF: Ribosome modulation factor
SD: Shine–Dalgarno
SSU: small subunit of the ribosome
tRNA: transfer RNA

## Introduction

### Ribosome hibernation is a cellular response to stress

**Figure 1:**
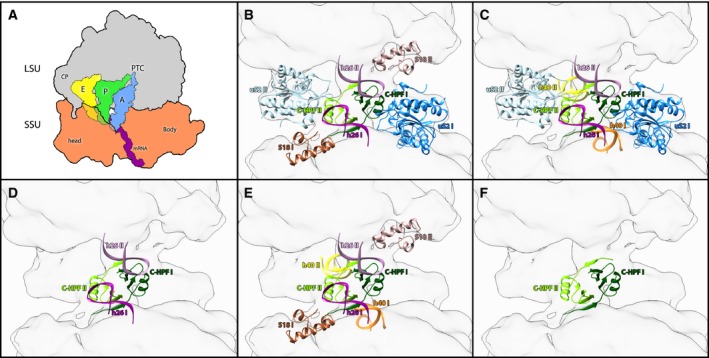
Species specificity of dimer interface of various bacterial systems. (A) Simplified scheme of the translating ribosome. The LSU in gray, SSU in orange, A‐site (A), P‐site (P) and E‐site (E) tRNA are colored in blue, green and yellow, respectively. The mRNA is marked in purple. The PTC, mRNA, central protuberance and the head and body domain in the SSU are also marked. (B–F) The specificity of dimer interface is demonstrated by highlighting the ribosomal components that form part of dimer interface of each species: *Bacillus subtilis, Staphylococcus aureus* 34 *, S. aureus* 33,* L. lectococcus and T. thermpophilus*, respectively. h26 is colored magenta‐sienna, h40 is colored orange‐yellow, C‐terminal HPF is colored green‐dark green, uS2 is colored blue‐light blue and s18 is colored brown‐rosy brown.

Proteins synthesis, which is performed by the ribosome in all living cells, is one of the most energy demanding cellular processes 1, 2, 3. Ribosomes, which are the universal multicomponent ribonucleoprotein assemblies that translate the genetic code into proteins, are comprised of two structurally independent subunits of unequal sizes that associate for creating the functioning ribosome. Within the active ribosome, the small subunit (SSU) binds the mRNA and provides the decoding site. The large subunit (LSU) contains the ribosomal catalytic site, namely the peptidyl transferase center (PTC), in which peptide bonds are being formed, and the exit tunnel through which the nascent proteins emerge out of the ribosome (Fig. 1A). The ability to control the pace of protein synthesis is essential for cell survival during the exponential phase as well as under stress conditions 4. One of such translation‐suppressing mechanism is the dimerization of two ribosomes into hibernation complexes (100S in prokaryotes and 110S in eukaryotes). This process benefits from the ribosomal internal flexibility, which is needed for protein synthesis processivity 5, and has been observed in bacterial, plastids 6, 7, 8, 9, 10 and mammalian cells 11. In most cells, it is facilitated by a hibernating promoting factor (HPF) 10, 12, 13. The importance of the dimer formation for cell survival has been demonstrated in several systems. Among them, HPF knock‐out in *Staphylococcus aureus* causes ribosome breakdown upon entering the stationary phase that correlates with the onset of cell death and attenuated virulence 14, 15. Deletion of HPF in *Lactococcus lactis* resulted in decreased viability after resuscitation from starvation conditions 8. Similarly, HPF‐depleted *Bacillus subtilis *cells' ability to regrow from the stationary phase has been decreased 12. In addition, deletion of *hpf* in *Listeria monocytogenes* impairs the survival of the bacteria in a murine model of infection 7 and compromises its tolerance to aminoglycosides 16. Ribosome hibernation is a reversible process. It has been shown that in *S. aureus*, 100S complexes are disassembled by a GTPase HflX under heat stress and a hitherto unknown major dissociation factor 17, 18. A minor, yet clinically important group of Gram‐negative bacteria (γ‐proteobacteria) that includes *Escherichia coli, Salmonella typhimurium*, *Yersinia pestis* and *Pseudomonas aeruginosa*, carries a shorter form of HPF (HPF^short^). In *E. coli*, HPF^short^ by itself does not induce dimer formation. Instead, it works in concert with the ribosome modulation factor (RMF) such that RMF (that is found only in species with HPF^short^) induces dimerization of two ribosomes, and forms immature 90S complex. Then the following binding of HPF converts 90S ribosomes to a mature, inactive 100S complex 19, 20, 21, 22, 23, 24. γ‐Proteobacteria also carry an additional protein, YfiA, which binds and inactivates 70S ribosomes 25, 26. YfiA is an antagonist to HPF^short^ and RMF by preventing 100S formation 19, 21. Early cryo electron microscopy (cryo‐EM) studies yielded structures of ribosomal dimers at rather low resolution, sufficient only to demonstrate that the two 70S ribosomes are bound to each other via their SSU and that the dimer interface has some degree of flexibility 8, 27, 28, 29.

## Results and Discussion

### Structural studies decipher species‐specific ribosome dimerization

Recent advances in cryo‐EM methods resulted in an abundance of detailed structures of the 100S complexes at near atomic resolutions (3.0–5.9 Å for an individual 70S composing the dimer and 4.1–11 Å for the whole dimer) 30, 31, 32, 33, 34, 35, 36. These demonstrated that in all systems dimer formation benefits from the ribosomal inherent flexibility. They also revealed not only fundamental differences between dimers that include HPF^short^ and RMF and those that include only a long version of HPF (HPF^long^) but also indicated both subtle and significant differences among the latter dimer type of the various bacterial species. Furthermore, the structures of the 100S hibernation complexes of *B. subtilis* 30, *S. aureus* 33, 34, *L. lactis* 32, and *Thermus thermophilus* 31 that carry HPF^long^ showed that this protein has two conserved functional domains [N‐terminal domain (NTD), and C‐terminal domain (CTD)] that are linked by a flexible linker. In all of the mentioned above structures, HPF^long^‐NTD is bound at the space between the ‘head’ and ‘body’ (Fig. 1A) of the SSU, near the subunit interface and adopts α/β fold (β1‐α1‐β2‐β3‐β4‐α2). The binding of HPF^long^‐NTD to the ribosome causes steric hindrance for transfer RNA (tRNA) and mRNA binding, thus inactivating the ribosome. HPF^long^‐NTD is stabilized in its pocket by interacting with the 16S rRNA and various ribosomal proteins. Once bound, HPF^long^‐NTD also overlaps with the binding sites of several antibiotics that bind the SSU (e.g. hygromycin B, tetracycline, edeine, kasugamycin, and pactamycin).

The HPF^long^‐CTD is bound to the interface between the two ribosomes comprising the dimer in all the structures mentioned above. One HPF^long^‐CTD creates a homodimer with the HPF^long^‐CTD of the second ribosome of the 100S complex. Mainly electrostatic interactions and domain swapping stabilize the HPF^long^‐CTD homodimer. Interestingly, the first β strand of one HPF^long^‐CTD crosses over and forms hydrophobic interactions with the last β strand of the other HPF^long^‐CTD (Fig. 1F). HPF^long^‐CTD homodimerization is the major contributor to the dimer formation as it is the single trait that is shared among all the 100S structures induced by HPF^long^.

In most 100S structures, the dimer interface was found to be further stabilized by additional inter‐ribosomal interactions (Fig. 1B–F). In *B. subtilis*, HPF^long^‐CTD of one ribosome further interacts with the N terminus of ribosomal protein bS18 of the second ribosome. In addition, the N‐terminal β‐hairpin and proximal region of the α2‐helix of protein uS2 establish a large interaction surface with the stem‐loop of helix h26 of the 16S rRNA 30. A similar interaction between uS2 and h26 was also observed in *S. aureus* 100S; however, in this structure bS18 does not seem to be involved in the dimer interface connections; instead, h40 interacts with HPF^long^‐CTD 34. Additionally, in another *S. aureus* 100S structure, the HPF^long^‐CTD does not interact with any other ribosomal components, whereas h26 of one ribosome interacts with its counter h26 instead of uS2 33. The finding that two independent studies on the same particle yielded different structure suggests that the 100S may adopt various conformations, thus further illustrating the flexibility of the dimer interface. On the other hand, it is plausible that this variation could result from the different experimental procedures employed for the creation of the dimer. In one study 34 the dimer was purified from a *S. aureus* strain carrying a high copy plasmid with the *hpf* gene, resulting in an increased amount of 100S ribosomes, whereas in the other study 33, a recombinant HPF was introduced to purified 70S ribosome at a 1 : 1 ratio. Similar to the first 100S structure mentioned above, in *L. lactis* the HPF^long^‐CTD of one ribosome is interacting with h40 (and probably uS18); however, in this structure h26 is not interacting with uS2. Instead it interacts with the h26 of the opposite ribosome 32, like in the second 100S structure. In *T. thermophilus*, 100S formation is solely attributed to HPF^long^‐CTD dimerization and not to any additional inter‐ribosome interactions 31. In most 100S dimer structures (excluding *T.* *thermophilus)*, h26 forms a part of the dimer interface. In* T. thermophilus*, h26 is shorter compared to h26 in the other species (the differences are 30 nucleotides in *S. aureus* and *B. subtilis*, 29 in *L. lactis* and 23 in *T. thermophilus*), thus h26 of one ribosome is located too far from any other ribosomal component of the other ribosome in the dimer and therefore cannot form any contacts that may contribute to the dimer stabilization.

The linker that connects the two domains of HPF^long^ could not be detected in most of the available 100S structures. This finding has been attributed to the flexibility of this linker. The sequences of both NTD and CTD domains of HPF^long^ are rather conserved among different bacteria, whereas the sequence and composition of the linker is hardy conserved 32. It was postulated that the linker might traverse the RMF binding site 30 thus interfering with proper helix formation between Shine–Dalgarno (SD) and anti‐SD sequence during translation initiation, hence causing inhibition of translation.

### Mechanisms for dimerization

**Figure 2:**
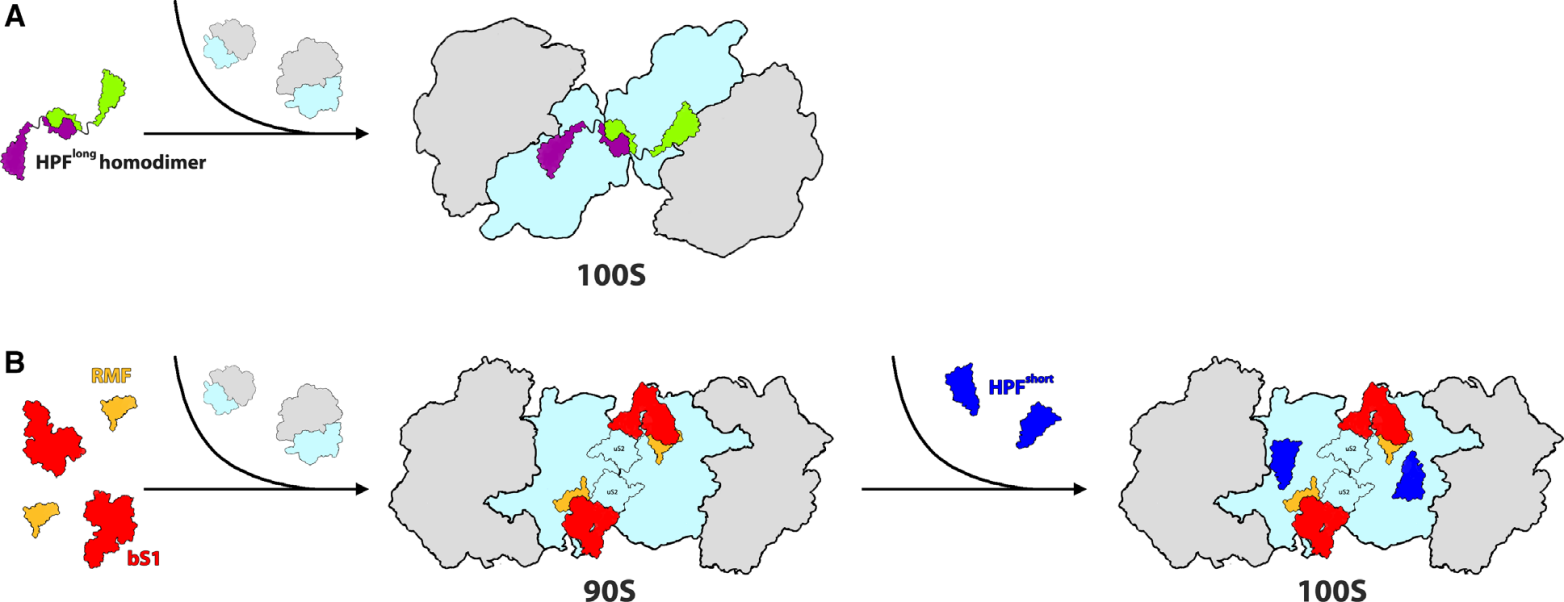
Overview of hibernation modes across bacteria. (A) 100S Ribosome formation is mediated by the binding of HPF^long^‐NTD (colored in green and purple to differentiate between each HPF molecule which binds to a different ribosome) to the ribosomal SSU (colored in light blue). Dimerization of the two ribosomes is facilitated by homodimerization of two HPF^long^‐CTDs. Secondary contacts may involve other ribosomal components, depending on the species. LSU is colored in gray. (B) Ribosome hibernation in *Escherichia coli* (and other γ‐proteobacteria). The initial binding of RMF and rProtein bS1 (colored in yellow and red, respectively) to the ribosome induces the formation of the immature 90S complex. Later HPF^short^ (colored in blue), binds at the catalytic site of the SSU (colored in light blue), resulting in the formation of the hibernating 100S dimer. LSU is colored in gray.

Long version of HPF is a homodimer in solution 10, 14, 30, 31, 32, 33, 34. Therefore, it has been suggested that within the cell environment, HPF^long^ is present at a dimeric state and the long linker of the dimeric HPF^long^ enables the HPF^long^‐NTD to interact with two independent 70S ribosomes, bringing them into close proximity (Fig. 2A). Then HPF^long^‐CTD that is in a dimeric form binds to the other ribosomal component at the 30S subunit rims (depending on the type of 100S complex) to further stabilize the ribosomal dimers 30, 31, 32. Alternatively, one cannot exclude the possibility that in the cell, HPF^long^ is a monomer and upon binding to the ribosome, dimerization occurs via homodimerization of the HPF^long^‐CTD.

The presence of HPF^long^ does not necessarily result in ribosome dimer formation, as demonstrated by the structures of ribosomes from *Mycobacterium smegmatis* 37, 38 (which is frequently used as a model of *Mycobacterium tuberculosis)* and the spinach chloro‐ribosome 35, in complex with HPF^long^. Both organisms carry HPF^long^; but ribosomal dimers have not been reported in these species. In both structures, the presence of ribosomal proteins S1 (bS1 in *M. smegmatis* and bS1c in spinach) is suggested to be hindering the dimer formation by steric clashes. Despite being the largest of the ribosomal proteins (61 kDa), only parts of bS1 have been observed in crystal structures and in cryo‐EM reconstructions of various structures of the SSU, due to its weak interactions with the ribosome 39, 40, 41, 42, 43. In the chloroplasts, bS1c is tightly associated with the chloro‐ribosome and *M. smegmatis* has an rRNA extension, H54a, a unique feature of mycobacteria, which seems to bind to bS1 and stabilize it, as well as H54a itself. Sequence comparison of HPF^long^‐CTD from *M. smegmatis*, *M. tuberculosis*, *S. aureus*, and *L. lactis* shows that one out of the five conserved residues proposed to be involved in the formation of 100S dimers is not conserved in Mycobacteria 32, 37. However, it is not clear that this is the reason that prevents 100S dimer formation in *M. smegmatis*. The structure of *M. smegmatis* in complex with HPF^long^ also suggests a small difference in the HPF^long^‐NTD binding site since it does not clash with the binding site of tRNA located at the ribosome’s exit site (E‐tRNA) (unlike the previously described dimers), as indicated by the existence of a density for the E‐tRNA that has been observed in the *M. smegmatis* cryo‐EM maps.

As previously mentioned, γ‐proteobacteria carry the HPF^short^ version and along with RMF, they induce the 100S dimer formation. HPF^short^ is very similar to HPF^long^‐NTD in terms of both sequence and structure, as demonstrated by the structure of *E. coli* 100S dimer 36. They also occupy the same binding pocket in all the structures mentioned above. However, in *E. coli,* HPF^short^ directly interacts with E‐site tRNA, similar to what has been observed in the *M. smegmatis* structure. Unlike HPF^short^, RMF shares minor similarities to HPF^long^‐CTD, which is bound at different locations on the ribosome. RMF interacts with both rRNA and proteins and it is located on the back of the small ribosomal subunit. RMF stabilizes a defined conformation of the 3' end of the 16S rRNA, which encompasses part of the anti‐SD sequence, similarly to the HPF^long^ linker. In the past, it was postulated that since neither HPF nor RMF is directly involved in the interactions of the two ribosomes, the dimer is formed due to conformational changes induced by the binding of these two proteins. This theory proved to be incorrect as no conformational changes, namely the swiveling of the SSU head, were detected. Instead, the cavity in which RMF is bound is capped by a large mass of additional density that was attributed to bS1. In the *E. coli* 100S structure, bS1 was found in an inactive, compacted conformation such that the C terminus of bS1 is folded back onto the 30S subunit, rather than extending into the solvent. The *E. coli* 100S is formed by two major bridges at the dimer interface where bS1 and uS2 from each 30S interact with uS4 and uS3, respectively, of the symmetry‐related SSU (Fig. 2B). Additionally, the C terminus of uS2 from one ribosome extends toward the mRNA entrance channel of its counterpart, thus effectively plugging the mRNA entrance channel, suggesting that such an interaction would not be possible on an already active translating ribosome to which mRNA is bound. The existence of two different mechanisms of ribosome dimerization (Fig. 2) and the conservation of protein uS2 as the main interaction partner support the idea that dimerization has been rescued in γ‐proteobacteria by the *rmf* gene upon loss of the CTD of HPF^long^ 32. Yet, it is still unclear why the presence of bS1 would promote dimer formation in γ‐proteobacteria, whereas in *M. smegmatis* and spinach that carry the HPF long version would hinder the dimerization. Perhaps the presence of the HPF^long^‐CTD does not allow the two ribosomes to bind to each other in a manner that is similar to *E. coli* 100S.

### Ribosome hibernation heterogeneity as a potential drug target

**Figure 3:**
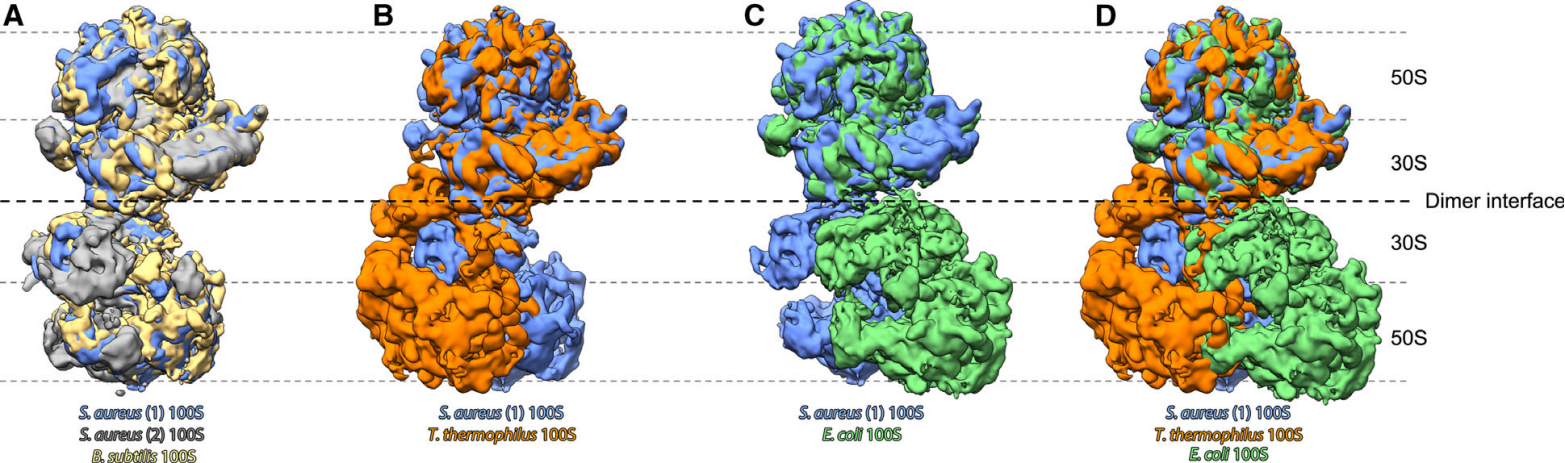
Conformations of 100S structures from different bacteria. Superposition of (A) *Bacillus subtilis* 100S (EMD‐3664, yellow) and two independently studied *Staphylococcus aureus* 100S (EMD‐3637, gray and EMD‐3638, blue) structures; (B) *Thermus thermophilus* 100S (colored orange) and *S. aureus* 100S (EMD‐3638, blue) structures; (C) *Escherichia coli* 100S (EMD‐0139, green) and *S. aureus* 100S (EMD‐3638, blue) structures; and (D) *T. thermophilus* 100S, *E. coli* 100S and *S. aureus* 100S (same coloring as A–C) structures. Both *S. aureus* 100S and *B. subtilis* 100S structures show a high degree of conformational conservation while *T. thermophilus* and *E. coli* 100S ribosomes are clearly staggered in a different conformation (thus, in B‐D, only one of the *S. aureus* 100S is shown as a representative of the second *S. aureus* 100S as well as *B. subtilis* 100S).

Structures of various HPF^long^‐mediated 100S ribosomes determined by cryo‐EM show a high degree of conformational homogeneity, with the exception of *T. thermophilus* 100S that is staggered slightly differently (Fig 3A,B). The reason for this is probably the lack of inter‐ribosomal interactions additional to the HPF^long^‐CTD homodimerization. In addition, head swiveling of the SSU, induced by HPF^long^ binding was observed only in one *S. aureus* study 34, benefiting from the inherent flexibility of the ribosomal SSU 44. In contrast, the HPF^long^‐mediated 100S dimers have markedly different conformations, compared to the *E. coli* 100S dimer (mediated by HPF^short^ and RMF) (Fig. 3C,D). Whereas *E. coli* 100S complex is created by ‘back‐to‐back’ interactions of the SSUs of each ribosome, the other HPF^long^‐mediated 100S complexes involve more ‘side‐to‐side’ (platform‐to‐platform) interactions of the SSU.

The HPF^long^‐NTD by itself is sufficient for silencing the ribosomes as well as HPF^short^ and RMF that are in fact not directly involved in the dimer interface. These structural findings raise the question: what is the biological significance of the ribosomal 100S dimer? As mentioned above, in *E. coli* 100S, the arrangement of the dimer allows for one N terminus of uS2 to block the mRNA entrance channel of the other ribosome. In addition, 100S dimers are less susceptible to degradation by RNases 39, 41, 43, 44. Nevertheless, the reason for the 100S formation is not well understood yet. Nevertheless, several hypotheses have been raised. Since 100S formation does not significantly alter the large rRNA surface exposed to RNases, it was suggested 30 that 100S formation may interfere with a specific ribosome degradation pathway, rather than preventing nonspecific RNase action on ribosomes. In addition, bacterial translation and transcription are coupled and the binding sites (e.g., uS2) of RNA polymerase on the 70S ribosome overlap with the 70S dimerization interface 40, 45, thus decreasing the translation activity 31, 34. Finally, proteins uS2, uS7, and uS11, along with rRNA helices 26 and 40, are part of the mRNA ‘platform binding center’ (PBC) 5, 17. This platform has been proposed to be a common site dedicated to mRNAs binding prior to the actual translation initiation to increase control over translation 5. uS2 was shown to possess a high level of flexibility and is involved in SSU dimerization in crystals 5. It takes active part in the dimer formation (as well as h26 and h40 in some of the 100S complexes reported above). Franken *et al* 32 propose that the interface of the dimer blocks the PBC by acting as a general, rather than a specific, inhibitor of mRNA translation initiation. Whether or not the 100S dimer formation does prevent protein synthesis in all or in some of the manners suggested above, it is clear that the hibernation complex manages this process by multiple mechanisms.

As the hibernation pathway is crucial for cell survival, it represents an attractive target for the development of novel antimicrobial agents. As different dimerization processes exist among different bacteria, including pathogens, this process may provide the basis for the species‐specific design of antimicrobial agents. Such potential inhibitors could interfere with HPF dimerization either by perturbing the function or of the critical flexible linker, or by competing with the HPF binding to the ribosomes. In addition, the stimulation of 100S disassembly factor activity may be used. Thus, bacterial growth inhibition by targeting 100S assembly or disassembly opens a new path for developing novel antibacterial therapy.

## Conflict of interest

The authors declare no conflict of interest.

## Author contributions

All authors participated in the interpretation of the results and in writing the article.
